# 

**Published:** 1893-03-25

**Authors:** 


### CONTENTS.

Hospital and Staff Liability for Negligence - - - 403

The Home Hospitals Association - - - - - - 404

The Virchow Banquet - - - - - - - - - 405

Contemporary Theory and Research - - - - - 406

The Hospital Clinic—

Royal Hospital for Diseases of the Chest, City Road, E.C.—Treatment of Pleurisy - - - - - - - 411

St. Mary's Hospital.—The Treatment of Pneumonia - - 411

Bristol Eye Hospital.—Treatment of Purulent Conjunctivitis - - - - - - - - - - - - 413

New Drugs, Appliances, and Things Medical.—"Dorina" Nursery Biscuits—Dr. Nansen and Bovril - - - - 414

The Practitioners' Bookshelf.—Orthopædy - - - - 414

Diet in Disease. By Mrs. Ernest Hart. XVII.—Gout - - 407

Annotations. — "Patients Conscientiously Treated" — The "Seamy Side" of "Home, Sweet Home"—The Children's Teeth 409

The Poor of London and their Medical Needs - - - 417

Notes and News - - - - - - - - - - 410

The Institutional Workshop—

Hospital Construction.—Radcliffe Infirmary, Oxford - - 415

Hospitals of the World.—The Progress of English Nursing - 416

The Poor of London and their Medical Needs - - - 418

Construction Notes - - - - - - - - - 418

Practical Departments.—Kitchen Ranges and Stoves - - 418

Scraps and Gleanings - - - - - - - - - 418

The Student of Medicine.—Appointments—Vacancies - - x

### EXTRA SUPPLEMENT.—THE NURSING MIRROR.

News from the Nursing World.—The East-end Mothers' Home—Our Fishermen—Health Talks—Practical Lecturing—More Cots Wanted—Common Sense—Moderate Remuneration—Good Health and Good Temper—Nurses for Norfolk—Accommodation for Accidents—A Queen's Nurse for Peebles—Odd Pennies—Nurse and Superintendent—Casualties at Chicago—Short Items—The Disabled Nurse—"The Hospital" Endowed Bed - - - - - - - - - - - cxci

Management of Consumption. VII.—Pneumothorax and Ulceration of Larynx - - - - - - - - - - cxciii

International Nursing Congress.—Suppressio Veri - - cxciii

A Nurse's Life in Canada - - - - - - - - cxciv

Nursing in Portugal—Appointments - - - - - cxcv

Minor Appointments - - - - - - - - - cxcv

Everybody's Opinion. — Linen Night-dresses — Soothing Syrups—Co-operation - - - - - - - - - cxcvi

Co operation or Competition? - - - - - - cxcvii

For Reading to the Sick.—Stepping Stones - - - - cxcvii

Where to Go - - - - - - - - - - - cxcvii

An Infallible Cough Mixture. A House Surgeon's Story cxcviii

Notes and Queries - - - - - - - - - - cxcviii

The Book World for Women and Nurses - - - - cxcix

